# The cost of wobble translation in fungal mitochondrial genomes: integration of two traditional hypotheses

**DOI:** 10.1186/1471-2148-8-211

**Published:** 2008-07-19

**Authors:** Xuhua Xia

**Affiliations:** 1 Department of Biology and Center for Advanced Research in Environmental Genomics, University of Ottawa 30 Marie Curie, Ottawa, K1N 6N5, Canada; 2 Ottawa Institute of Systems Biology, University of Ottawa, Ottawa, Canada

## Abstract

**Background:**

Fungal and animal mitochondrial genomes typically have one tRNA for each synonymous codon family. The codon-anticodon adaptation hypothesis predicts that the wobble nucleotide of a tRNA anticodon should evolve towards maximizing Watson-Crick base pairing with the most frequently used codon within each synonymous codon family, whereas the wobble versatility hypothesis argues that the nucleotide at the wobble site should be occupied by a nucleotide most versatile in wobble pairing, i.e., the tRNA wobble nucleotide should be G for NNY codon families, and U for NNR and NNN codon families (where Y stands for C or U, R for A or G and N for any nucleotide).

**Results:**

We here integrate these two traditional hypotheses on tRNA anticodons into a unified model based on an analysis of the wobble costs associated with different wobble base pairs. This novel approach allows the relative cost of wobble pairing to be qualitatively evaluated. A comprehensive study of 36 fungal genomes suggests very different costs between two kinds of U:G wobble pairs, i.e., (1) between a G at the wobble site of a tRNA anticodon and a U at the third codon position (designated M_U3:G_) and (2) between a U at the wobble site of a tRNA anticodon and a G at the third codon position (designated M_G3:U_).

**Conclusion:**

In general, M_U3:G _is much smaller than M_G3:U_, suggesting no selection against U-ending codons in NNY codon families with a wobble G in the tRNA anticodon but strong selection against G-ending codons in NNR codon families with a wobble U at the tRNA anticodon. This finding resolves several puzzling observations in fungal genomics and corroborates previous studies showing that U3:G wobble is energetically more favorable than G3:U wobble.

## Background

The wobble versatility hypothesis [1-6], abbreviated as WVH, states that the wobble site of tRNA anticodon should have G for NNY codons (where Y stands for C or U and N for any nucleotide) because G can pair with both C and U in RNA, and should have U for NNR to pair with both A and G. For NNN codon families, the wobble site should be U because U is known to be the most versatile in wobble-pairing [7-12]. In contrast, the codon-anticodon adaptation hypothesis, or CAAH for short, invokes the codon usage bias as a determining factor, i.e., the wobble site of tRNA anticodon should co-evolve with codon usage so that the nucleotide in the wobble site of tRNA anticodon should match the most abundant codon in a synonymous codon family [6,13-15]. The association between the major codon and the anticodon of the most abundant tRNA has been documented in *Escherichia coli *[16,17], *Saccharomyces cerevisiae *[18], and other species and organelles [15,19-22].

Here we develop a general hypothesis of codon-anticodon adaptation based on an analysis of wobble costs, and derive its predictions that can be tested by genomic data. The wobble cost may be viewed as reduction in decoding efficiency and accuracy because such reduction would be selected against over evolutionary time. We will refer to this new general hypothesis based on wobble cost as WCH (for wobble cost hypothesis). The two traditional hypotheses, CAAH and WVH, will be shown to be special forms of WCH.

Following the shorthand notation of Ogle et al. [23], I designate the translation cost through wobble base-pairing between nucleotide i at the third codon position of a codon and the nucleotide j at the wobble site of tRNA anticodon as M_i3:j _(where M is for wobble cost. The letter C would be more suitable to represent cost but it may confuse with the nucleotide C). We assume M_i3:j _= 0 if nucleotides i and j form Watson-Crick base pairing. The reason for this assumption is that C:G and A:U pairs have not been found to contribute to ribosomal stalling (which reduces translation efficiency) or amino acid mis-incorporation (which reduces translation accuracy) although almost all non-Watson-Crick base pairings have been shown to reduce translation efficiency and accuracy. We define M_Y3:U _as the wobble cost between a wobble U at the tRNA anticodon and a C or U at the third codon position. For simplicity, we also assume M_A3:A _= M_C3:A _= M_G3:A _= M_A3:C _= M_C3:C _= M_U3:C _= M_A3:G _= M_G3:G _= M_O _(where the subscript O is for "other", i.e., M_O _is the wobble cost between base pairs that are not Watson-Crick base pairs, not U3:G or G3:U base pairs and not Y3:U pairs). In general, M_U3:G _and M_G3:U _are expected to be smaller than M_C3:U_, M_U3:U_, and M_O _because G and U can base-pair in RNA, M_Y3:U _is expected to be smaller than M_O _because a wobble U at tRNA anticodon is known to be the most versatile in wobble-pairing [7-12].

A classical study of U:G wobble pairs [24] suggests a preference for the U being at the 3' end rather than at the 5' end, which implies that U3:G wobble pair is energetically more favorable than G3:U wobble pair [25]. Subsequent studies have shown that, while the U3:G wobble pair occurs on the ribosome, the unmodified G3:U wobble pair does not [2,23,26]. These findings suggest that M_U3:G _may be smaller than M_G3:U_, although its generality is unknown.

### Two-fold NNY and NNR codon families

First consider the NNY codon family where Y is either C or U. Designate the number of C-ending and U-ending synonymous codons by N_C _and N_U_, respectively, and the total cost of wobble pairing as M_wG _when the wobble site of the anticodon is G, and as M_wA _when the wobble nucleotide is A (we do not need to consider the case when the wobble site is U or C for NNY codon families because such cases have never been observed and because a tRNA with a wobble U or C to translate NNC and NNU codons is against physiochemical reasons). We now express the total cost M_wG _and M_wA _as

(1) $$\begin{array}{l} M_{wG}=N_{U}M_{U3:G}\\ M_{wA}=N_{C}M_{C3:A}=N_{C}M_{O} \end{array}$$

**Figure 1:**
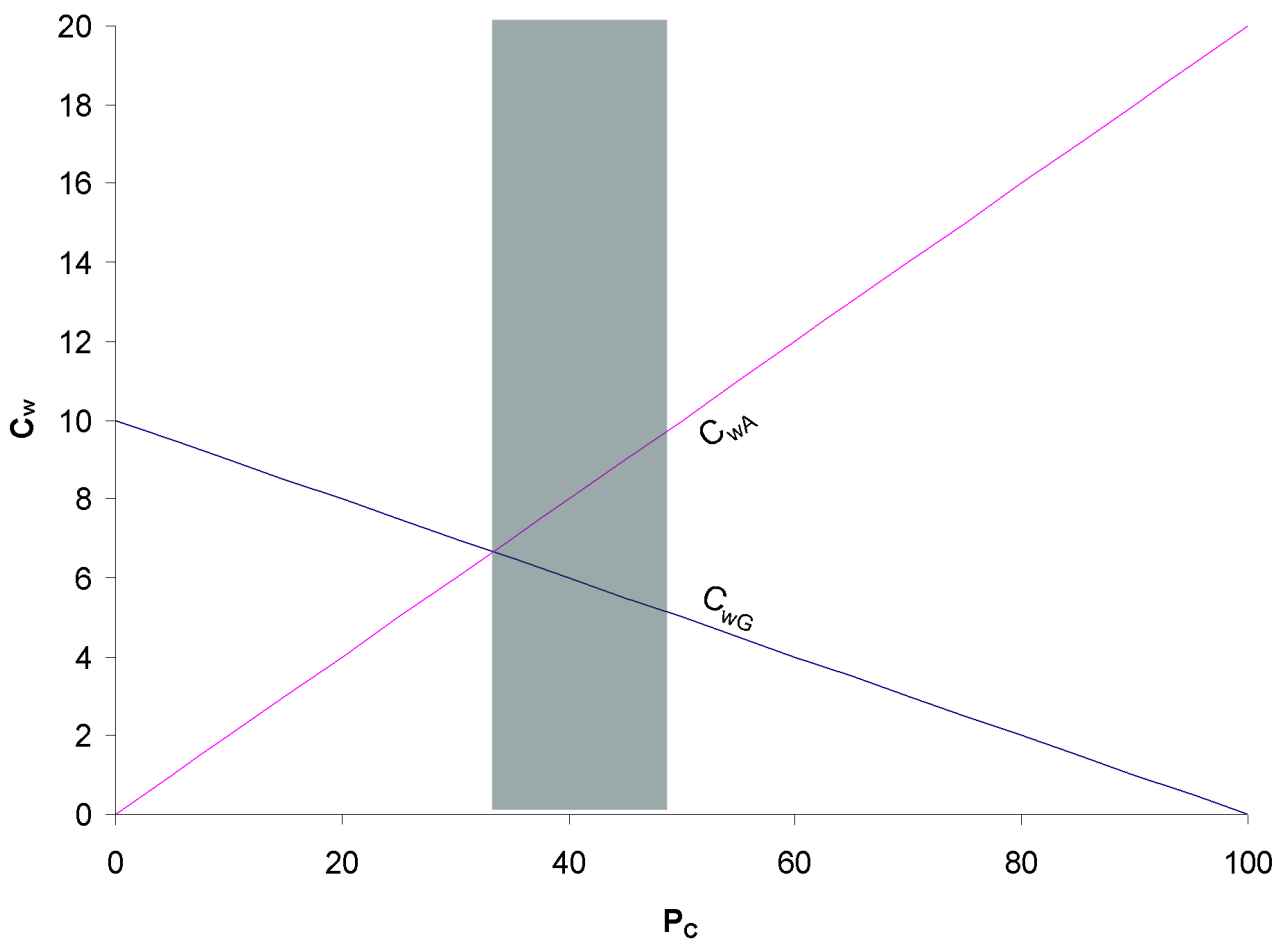
Conceptual illustration of the dependence of wobbling cost involving a G or an A at the wobble site of tRNA anticodon (M_wG _and M_wA_) on the proportion of C-ending codons (P_C_) in an NNY codon family, with M_C3:A _= 2M_U3:G_. The shaded area corresponds to P_C _smaller than 1/2 but larger than 1/3.

Note that M_C3:G _= M_U3:A _= 0 according to our definition. The dependence of M_wG _and M_wA _on the relative frequencies of N_C_, expressed as proportion of C-ending codons in the NNY codon family (P_C_), is graphically shown in Fig. 1, with M_O _assumed to equal 2•;M_U3:G_. The condition for M_wG _= M_wA_, i.e., when the wobble site of the tRNA anticodon can take either G or A without a fitness differential, is

(2) $$\frac{M_{U3:G}}{M_{O}}=\frac{N_{C}}{N_{U}}=\frac{P_{C}}{1-P_{C}}$$

In the scenario in Fig. 1 with M_O _= 2M_U3:G_, the condition for M_wG _= M_wA _is P_C _= 1/3. Thus, when P_C _drifts above 1/3, it would become more beneficial (less costly) to have a G at the wobble nucleotide of tRNA anticodon. However, when PC drifts below 1/3, then a wobble A would be more advantageous and should be favored by natural selection. Naturally, if M_U3:G _= 0, then we should always have a wobble G at the tRNA anticodon regardless of codon usage.

Now we consider three special cases with reference to Fig. 1 and the two traditional hypotheses, CAAH and WVH. First, if N_C _= N_U _= N/2, then CAAH, which predicts that the wobble nucleotide of tRNA anticodon should form Watson-Crick base pair with the most abundant codon, has no prediction at all because the two codons are equally abundant. In contrast, if we assume M_U3:G _< M_O _as is depicted in Fig. 1, we have M_wG _< M_wA_, and WCH predicts that the anticodon wobble site should be a G. This is the same prediction as WVH.

Second, if N_C _> N_U_, then M_wG _< M_wA _assuming M_U3:G _≤ M_O_, and WCH predicts that the anticodon wobble site should be occupied by a G. This prediction is shared by both CAAH and WVH.

Third, when N_C _< N_U _but N_C _> N_U_·M_U3:G_/M_O_, then WCH will still predict a G at the wobble nucleotide of tRNA anticodon because M_wG _is still smaller than M_wA_. Take the scenario in Fig. 1 for example, when N_C _< N_U _but P_C _> 1/3 (which correspond to the shaded area in Fig. 1), we have M_wG _< M_wA _(Fig. 1), so natural selection should favor a G at the wobble site of tRNA anticodon. WVH happens to have the same prediction. However, CAAH will predict an A at the wobble site of tRNA anticodon if U-ending codons are more abundant than C-ending codons. This is in contrast to WVH, i.e. CAAH is inapplicable when P_C _is within the shaded range in Fig. 1.

Fourth, when N_C _<< N_U_, especially in the extreme case when N_C _= 0, then Eq. (1) is reduced to M_wG _= N_U• _M_U3:G _and M_wA _= 0. Because now M_wG _> M_wA_, WCH predicts an A at the anticodon wobble site. In this case, WVH would still predict a G at the wobble site because it ignores the codon frequencies (i.e., it ignores the relative magnitude of N_C _and N_U_), but CAAH would predict an A at the wobble site, which is the same prediction as WCH. Only in this particular case when N_C _<< N_U _can CAAH and WVH be clearly differentiated. As is depicted in Fig. 1, the chance for CAAH to be supported decreases as M_U3:G _decreases relative to M_O_. CAAH would have little chance to be unambiguously supported if M_U3:G _is close to 0. Thus, as long as M_U3:G _is small relative to M_O_, we should expect CAAH to be less supported than WVH. A recent study shows that CAAH indeed receives much less support than WVH when both were tested against fungal mitochondrial genomic data [27].

The simple Eq. (1) offers a way to assess relative costs of different wobble pairs. For example, if nature has chosen a G at the wobble site of the tRNA anticodon, then we may infer that M_wG _< M_wA_. So, from Eq. (1), we have

(3) $$\begin{array}{l} N_{U}M_{U3:G}<N_{C}M_{O}\\ \frac{M_{U3:G}}{M_{O}}<\frac{N_{C}}{N_{U}} \end{array}$$

Suppose we have an NNY codon family translated by a tRNA with a G at the wobble site of the anticodon. If M_U3:G _is large, then there would be strong selection favoring C-ending codons against U ending codons. So the ratio of N_C_/N_U _may be very large. In such a case the estimated M_U3:G_/M_O _ratio, being smaller than a very large value, is not informative. However, in different codon families, mutation pressure may allow N_U _to drift up relative to N_C_. Suppose we have three NNY codon families each translated by a tRNA with a G at the anticodon wobble site. If the N_C_/N_U _ratio for the three NNY codon families are 100/2, 80/100 and 200/20, respectively, we may infer that M_U3:G_/M_O _< 80/100 (because it is the most precise). Alternatively, with many N_C_/N_U _ratios from many NNY codon families translated by tRNA with a G at its anticodon wobble site, we may compute the lower 95% confidence limit of the N_C_/N_U _ratio (LCL_95.G _where the subscript G indicates tRNA with a G at the anticodon wobble site) and infer that M_U3:G_/M_C3:A _< LCL_95.G_.

If we always have very large N_C_/N_U _ratios, we may infer that selection against U-ending codons must be strong, with little chance for mutation to elevate N_U_. This is a strong indication of a large M_U3:G_. Along the same line of reasoning, we may infer that M_U3:G _is very small if N_U _can often as large as, or even larger than, N_C_.

Similarly, if nature has chosen an A at the wobble site of tRNA anticodon, then we may infer M_wG _> M_wA_, so

(4) $$\begin{array}{l} N_{U}M_{U3:G}>N_{C}M_{O}\\ \frac{M_{U3:G}}{M_{O}}>\frac{N_{C}}{N_{U}} \end{array}$$

We can apply exactly the same rationale for the NNR codon family leading to parallel conclusions. For example, if nature has chosen a U at the wobble site of the tRNA anticodon, then we may infer that M_wU _< M_wC_, so that

(5) $$\frac{M_{G3:U}}{M_{O}}<\frac{N_{A}}{N_{G}}$$

Similarly, if M_G3:U _is very large, then there should be strong selection against G-ending codons in favor of A-ending codons. This will produce large N_A_/N_G _ratios. In contrast, a large N_G _comparable to N_A _indicates a very small M_G3:U _cost.

Given previous studies indicating that U3:G is energetically much more favorable than G3:U [2,23-26], we should expect M_U3:G _< M_G3:U_. The reasoning above paves the way for us to test whether this is generally true among the genetically diverse fungal species.

### Three-fold AUH (Isoleucine) codons

Designate codons ending with A, C, and U as N_A_, N_C _and N_U_, respectively. The wobble cost of having an A, G, C, or U at the wobble site of tRNA anticodon is

(6) $$\begin{array}{l} M_{wA}=N_{A}M_{A3:A}+N_{C}M_{C3:A}=(N_{A}+N_{C})M_{O}\\ M_{wG}=N_{A}M_{A3:G}+N_{U}M_{U3:G}=N_{A}M_{O}+N_{U}M_{U3:G}\\ M_{wC}=N_{A}M_{A3:C}+N_{C}M_{C3:C}+N_{U}M_{U3:C}=(N_{A}+N_{C})M_{O}+N_{U}M_{U3:C}\\ M_{wU}=N_{C}M_{C3:U}+N_{U}M_{U3:U}=(N_{C}+N_{U})M_{Y3:U} \end{array}$$

It is obvious that M_wC _is always greater than M_wA_, so we should never find a C at the wobble site of a tRNA^Ile ^anticodon, i.e., we can disregard M_wC_. If nature has chosen G at the wobble site of tRNA^Ile ^anticodon, then we may infer that M_wG _is the smallest. From M_wG _< M_wA _and M_wG _< M_wU_, we have

(7) $$\begin{array}{l} \frac{M_{U3:G}}{M_{O}}<\frac{N_{C}}{N_{U}}\\ N_{U}M_{U3:G}<(N_{C}+N_{U})M_{X3:U}-N_{A}M_{O}\\ \frac{M_{U3:G}}{M_{Y3:U}}<\frac{N_{C}+N_{U}-N_{A}}{N_{U}},\text{assuming }M_{Y3:U}<M_{O} \end{array}$$

where the assumption is made on the basis of previous observations that U is generally the most versatile in wobble-pairing among the four nucleotides [7-12].

### Four-fold NNN codons

Designate the number of codons ending with A, C, G, and U as N_A_, N_C_, N_G_, and N_U_, respectively. The wobble costs involving an A, C, G or U at the wobble site of tRNA anticodon are, respectively,

(8) $$\begin{array}{l} M_{wA}=N_{A}M_{A3:A}+N_{C}M_{C3:A}+N_{G}M_{G3:A}=(N_{A}+N_{C}+N_{G})M_{O}\\ M_{wC}=N_{A}M_{A3:C}+N_{C}M_{C3:C}+N_{U}M_{U3:C}=(N_{A}+N_{C})M_{O}+N_{U}M_{U3:C}\\ M_{wG}=N_{A}M_{A3:G}+N_{G}M_{G3:G}+N_{U}M_{U3:G}=(N_{A}+N_{G})M_{O}+N_{U}M_{U3:G}\\ M_{wU}=N_{C}M_{C3:U}+N_{G}M_{G3:U}+N_{U}M_{U3:U}=(N_{C}+N_{U})M_{Y3:U}+N_{G}M_{G3:U} \end{array}$$

If nature has chosen a U at the wobble site of the tRNA anticodon, then Eq. (8) does not give us any simple inequality to estimate the cost ratios. However, if nature has chosen an A at the wobble site of the tRNA anticodon, then from M_wA _< M_wG _and M_wA _< M_wC_, we can infer

(9) $$\begin{array}{l} \frac{M_{U3:G}}{M_{O}}>\frac{N_{C}}{N_{U}}\\ \frac{M_{U3:C}}{M_{O}}>\frac{N_{G}}{N_{U}} \end{array}$$

In what follows, we estimate M_U3:G_/M_O_, M_G3:U_/M_O_, and M_U3:C_/M_O _by using fungal mitochondrial genomic data. Cells in fungal species are generally rapid-replicating which necessitates efficient translation. Rapidly replicating unicellular organisms are theoretically expected to be under strong selection to increase the rate of biosynthesis [15,28] and they typically exhibit strong codon-anticodon adaptation [29]. Thus, fungal species should be ideal for evaluating evolutionary hypothesis on codon-anticodon adaptation.

## Methods

We retrieved 36 fungal mitochondrial genomes (Table 1) by using NCBI Entrez. Three different genetic codes are used in different fungal genomes. Among the 36 fungal mitochondrial genomes, seven genomes use translation table 3, 27 genomes use translation table 4, and two genomes use translation table 16 (Table 1). When results are similar among genomes using the same translation table, only results from a representative genome are presented. The number of codon families supporting CAAH (N_CAAH_) and WVH (N_WVH_) is compiled following the following rationale [27]. Suppose a lysine (Lys) codon family has 20 AAA and 60 AAG codons. WVH would ignore the codon usage bias and predict a wobble U in the tRNA^Lys ^anticodon because U can pair with both A and G, whereas CAAH would predict a wobble C in the tRNA^Lys ^anticodon to maximize the Watson-Crick match with the more frequent G-ending codons. If the tRNA^Lys ^anticodon is found to have a wobble U, then WVH is supported; if a wobble C is found, then CAAH is supported. If we have 60 AAA codons and 20 AAG codons and if tRNA^Lys ^anticodon has a wobble U, then both hypotheses are supported, i.e., they are indistinguishable and are not included in Table 1. The methionine codon families are not included in Table 1 but discussed in detail elsewhere [27,30].

**Table 1:** Number of codon families unambiguously supporting the codon-anticodon adaptation hypothesis (N_CAAH_) and the wobble versatility hypothesis (N_WVH_) in each fungal species.

Species	Accession*	**Code**^†^	N_CAAH_	N_WVH_
*Allomyces macrogynus*	NC_001715	4	1	13
*Ashbya gossypii ATCC 10895*	NC_005789	3	0	10
*Aspergillus niger*	NC_007445	4	0	12
*Aspergillus tubingensis*	NC_007597	4	0	12
*Candida albicans SC5314*	NC_002653	4	1	11
*Candida glabrata*	NC_004691	3	0	12
*Candida metapsilosis*	NC_006971	4	1	15
*Candida orthopsilosis*	NC_006972	4	1	15
*Candida parapsilosis*	NC_005253	4	1	15
*Candida stellata*	NC_005972	4	1	13
*Moniliophthora perniciosa*	NC_005927	4	0	13
*Epidermophyton floccosum*	NC_007394	4	2	12
*Harpochytrium sp. JEL94*	NC_004760	4	1	3
*Harpochytrium sp. JEL105*	NC_004623	4	1	4
*Hyaloraphidium curvatum*	NC_003048	4	1	4
*Hypocrea jecorina*	NC_003388	4	1	13
*Kluyveromyces lactis*	NC_006077	4	0	13
*Kluyveromyces thermotolerans*	NC_006626	3	0	11
*Lecanicillium muscarium*	NC_004514	4	0	10
*Monoblepharella sp. JEL 15*	NC_004624	4	0	15
*Mortierella verticillate*	NC_006838	4	1	13
*Penicillium marneffei*	NC_005256	4	1	12
*Pichia Canadensis*	NC_001762	4	2	12
*Podospora anserine*	NC_001329	4	0	13
*Rhizophydium sp.136*	NC_003053	16	0	2
*Rhizopus oryzae*	NC_006836	4	1	14
*Saccharomyces cerevisiae*	NC_001224	3	1	12
*Saccharomyces castellii*	NC_003920	3	0	14
*Saccharomyces servazzii*	NC_004918	3	0	9
*Schizophyllum commune*	NC_003049	4	1	13
*Schizosaccharomyces japonicus*	NC_004332	4	1	16
*Schizosaccharomyces octosporus*	NC_004312	4	1	14
*Schizosaccharomyces pombe*	NC_001326	4	1	14
*Smittium culisetae*	NC_006837	4	1	15
*Spizellomyces punctatus1*	NC_003052	16	0	4
*Yarrowia lipolytica*	NC_002659	3	0	11
Sum			23	414

* GenBank accession number.

^† ^Genetic code.

The tRNA and CDS sequences were extracted and analyzed by using DAMBE [31,32]. The CDS-derived codon usage is also computed with DAMBE. The anticodon in almost all tRNA sequences from all species share the regular feature of being flanked by two nucleotides on either side to form a loop that is held together by a stem. For example, the anticodon loop (AC loop) of the tRNA^Arg ^genes translating CGN codons in *Epidermophyton floccosum *is 28CGUG**UU**ACG**GC**CACG42, where the starting and ending numbers indicate the position of the AC loop in the tRNA sequence, with the anticodon 5'-ACG-3' (matching codon CGU) flanked by two nucleotides on either side (in bold) to form a loop that is held together by a stem made of the first and the last four nucleotides. Similarly, the other tRNA^Arg ^translating AGR codons is 25AAAAUA**CU**UCU**AA**UAUUUU43, with the AC loop held together by a six-base stem. However, some tRNA sequences have a suspicious AC loop and DAMBE will flag them out. The AC loop is then identified by aligning the tRNA sequences against other isoaccepting tRNA sequences with a regular AC loop [6]. Some tRNA anticodon loop has the anticodon flanked by three instead of two nucleotides. For example, the anticodon loop in tRNA^Leu ^in the mitochondrial genome of *Kluyveromyces thermotolerans *is GAUAC**UCU**UAA**GAU**GUAUU, with the anticodon UAA flanked by three nucleotides (in bold) on both sides. There are a few tRNA sequences in which anticodon loop cannot be identified.

Some mitochondrial genomes in GenBank are annotated incorrectly. For example, tRNA^Pro ^in the mitochondrial genome of *Ashbya gossypii *ATCC 10895 has an anticodon of UGG matching codon CCA (the most frequently used proline codon), but the GenBank file (NC_005789) annotated the anticodon to match codon CCU.

A few fungal mitochondrial genomes do not have a complete set of tRNA genes. For example, the mitochondrial genomes of *Hyaloraphidium curvatum *and *Harpochytrium sp*. JEL94 have seven and eight tRNA genes, respectively, and consequently will need tRNA import from the nuclear genome. This may cause complication in analyzing codon-anticodon adaptation. However, removing such genomes does not alter the conclusions.

Some species exhibit extreme avoidance of certain codon families. For example, *Ashbya gossypii *ATCC 10895 codes Arg with only AGR codons without using any CGN codons. In contrast, *Hyaloraphidium curvatum *codes Arg with only CGN codons without using any AGR codons. Such avoidance of certain codon families would facilitate the evolutionary loss of the associated tRNA [33-35], although it is not always clear whether the avoidance is the cause or the consequence of the loss of the associated tRNA.

We computed relative synonymous codon usage, or RSCU [36], as a measure of codon usage bias within a codon family by using DAMBE [31,32]. Some coding sequences are incomplete. For example, the cox1 CDS in *Aspergillus niger *is annotated as "join(<19768..20614,21640..22495)". The first two nucleotides (i.e., at positions 19768 and 19769) represent a partial codon and are discarded in computing codon frequencies.

## Results and discussion

### Wobble cost between G and U: MU3:G and MG3:U

Recall that the two inequalities

(10) $$\begin{array}{l} \frac{M_{U3:G}}{M_{O}}<\frac{N_{C}}{N_{U}}\text{ and}\\ \frac{M_{G3:U}}{M_{O}}<\frac{N_{A}}{N_{G}} \end{array}$$

are, respectively, for NNY codons translated by tRNA with a G at the wobble site of tRNA anticodon, and for NNR codons translated by tRNA with a U at the wobble site of tRNA anticodon. The observed N_C_/N_U _ratios for *Allomyces macrogynus *(representing fungal mitochondrial genomes with translation table 4) are much smaller than N_A_/N_G _ratios (Table 2). The smallest N_C_/N_U _value is 0.279 whereas the smallest N_A_/N_G _value is 2.372 (Table 2). We have mentioned before that, if M_U3:G _is very small, then a wobble G at the tRNA anticodon will not impose strong selection against U-ending codons, and N_U _may drift up and down with mutation relative to N_C_. This will lead to relatively small N_C_/N_U _ratios. From the minimum N_C_/N_U _value of 0.279, we may infer that M_U3:G _< 0.279•M_O_, i.e., M_U3:G _is quite small relative to M_O_.

**Table 2:** N_C_/N_U _ratios for NNY codons and N_A_/N_G _ratios for NNR codons in *Allomyces macrogynus *(representing fungal mitochondrial genomes with translation table 4).

CF*	AA^†^	AC^‡^	N_Cod _^§^	N_C_/N_U_	CF*	AA^†^	AC^‡^	N_Cod _^§^	N_A_/N_G_
AAY	N	GUU	360	0.295	AAR	K	UUU	411	2.543
AGY	S	GCU	230	0.314	AGR	R	UCU	202	6.769
CAY	H	GUG	213	0.357	CAR	Q	UUG	173	3.806
GAY	D	GUC	356	0.299	GAR	E	UUC	263	2.372
UAY	Y	GUA	390	0.279	UUR	L	UAA	742	5.870
UGY	C	GCA	94	0.382					
UUY	F	GAA	592	0.726					

Minimum				0.279					2.372
Mean				0.379					4.272
Std Dev				0.157					1.975

* CF: Codon family

^† ^AA: One-letter code of amino acid

^‡ ^AC: Anticodon

^§ ^N_Cod_: Number of codons in the codon family

The AUH codon family coding for amino acid Ile in *A. macrogynus *mitochondrial genome is translated by a tRNA with a GAU anticodon. According to Eq. (7), the M_U3:G_/Co ratio should also be smaller than the N_C_/N_U _ratio. The observed N_C_/N_U _ratio is 0.3605 (= 159/441). This is similar to the N_C_/N_U _ratio in NNY codon families (Table 2). Thus, the wobble cost of M_U3:G _relative to M_O _from the AUH codon family is similar to that derived from NNY codon families.

The CUN codon family coding for amino acid Leu in *A. macrogynus *mitochondrial genome is translated by a tRNA with an AAG anticodon. Note that no A→I conversion has been observed in mitochondria [37,38] so Eq. (9) is applicable. According to Eq. (9), the M_U3:G_/Co ratio should be greater than the N_C_/N_U _ratio. The observed N_C_/N_U _ratio is 0.1905 (= 48/252). Thus we have M_U3:G _> 0.1905•M_O_. This inequality, together with the previous inequality of M_U3:G _< 0.279•M_O_, leads to 0.1905•M_O _< M_U3:G _< 0.279•M_O_.

Given that M_U3:G _> 0.1905•M_O_, we can infer that M_wG _> M_wA _when N_C_/N_U _< 0.1905, i.e., when U-ending codons are more than five times as frequent as C-ending codons. In other words, when N_C_/N_U _< 0.1905, the wobble cost of having an A at the wobble site of the tRNA anticodon is smaller than that of having a G at the wobble site, and should be favored by natural selection. Among the 36 fungal mitochondrial genomes, only two genomes have a NNY codon family with a correspondent tRNA that has an A at the wobble site of its anticodon. In the mitochondrial genome of *Penicillium marneffei*, the tRNA translating the AAY codon family has a wobble A in its anticodon. The N_C_/N_U _ratio is 0.087 (= 41/473) which is much smaller than 0.1905. Similarly, in the mitochondrial genome of *Pichia Canadensis*, the tRNA translating the AGY codon family has a wobble A in its anticodon. The N_C_/N_U _ratio is 0.0083 (= 1/120) which is also much smaller than 0.1905. Thus, the prediction that a wobble A at the tRNA anticodon is advantageous over a wobble G when N_C_/N_U _< 0.1905 is consistent with empirical data.

In contrast to the small N_C_/N_U _ratios in NNY, AUH and CUN codon families, all N_A_/N_G _ratios in NNR codon families are substantially larger (Table 2). We have argued before that, if M_G3:U _is very small, then a U at the wobble site of tRNA anticodon would impose little selection against G-ending codons in NNR codon families, and mutation may allow N_G _to drift up, leading to large N_G _values relative to N_A_. However, if M_G3:U _is large, then G-ending codons should be strongly selected against and N_G _would be small relative to N_A_, leading to large N_A_/N_G _ratios. The much larger N_A_/N_G _ratios than N_C_/N_U _ratios (t = 5.2967, DF = 10, p = 0.0003, two-tailed test) suggest that M_G3:U _is much greater than M_U3:G_.

There is a caveat in evaluating the relative magnitude of M_U3:G_/M_O_, and M_G3:U_/M_O _by the N_C_/N_U _and N_A_/N_G _ratios because these ratios can be affected by AT-biased mutations. The mitochondrial genome of *A. macrogynus *is 57472 bp, with the number of C+G being 22700 and that of A+U being 34772. If we exclude those nucleotides in coding sequences, then the numbers of C+G and A+U are 14136 and 17656, respectively. This may be considered as the background frequencies maintained by mutation bias, which leads to the expected N_C_/N_U _ratio of 0.8001 (=N_C+G_/N_A+U_) and that of N_A_/N_G _ratio of 1.2490 (=N_A+U_/N_C+G_). Thus, we expect N_C_/N_U _to be smaller than N_A_/N_G _even when there is no difference between M_U3:G _and M_G3:U_. To establish the argument that M_U3:G _is indeed smaller than M_G3:U_, we need to demonstrate that (1) there is no selection against U-ending codons in NNY codon families by showing that the observed N_C_/N_U _ratio is not greater than 0.8001, and (2) there is selection against G-ending codons in NNR codon families by showing that N_A_/N_G _is significantly greater than 1.2490. It is not enough to show that N_C_/N_U _< N_A_/N_G_.

We note that the observed N_C_/N_U _values for the seven NNY codon families in Table 2 are all smaller than the expected 0.8001, suggesting no selection against U-ending codons in NNY codon families (i.e., small M_U3:G_). In contrast the observed N_A_/N_G _ratios for the five NNR codon families are all much greater than the expected 1.2490 (Table 2), consistent with the interpretation of selection against G-ending codons in NNR codon families (i.e., large M_G3:U_). This is consistent with the interpretation that M_U3:G _< M_G3:U_. One can perform a χ^2^-test for each of the NNR codon families to see if G-ending codons are underused. The tests are all highly significant, with p < 0.00001.

The results are similar for fungal genomes with translation table 3, with the result from a representative species (*Saccharomyces cerevisiae*) presented in Table 3. Again the N_C_/N_U _ratios in NNY codon families are much smaller than N_A_/N_G _ratios in NNR codon families. We should note that the *S. cerevisiae *mitochondrial genome is much more AT-biased than the *A. macrogynus *mitochondrial genome, with the proportion of (G+C) in non-coding sequences being only 0.1484. The reason for the GC deficiency in yeast is not clear, but it may be caused either by mutation bias or by the low abundance of C in living cells [39-41]. In any case, the expected N_C_/N_U _and N_A_/N_G _ratios, given the biased genomic AT content, are 0.1742 and 5.7405, respectively. We note that the observed N_C_/N_U _ratios among the NNY codon families are all smaller than the expected value of 0.1742 except for the UUY (Phe) codon family (Table 3), against suggesting little selection against U-ending codons (i.e., small M_U3:G_). In contrast, the N_A_/N_G _ratios are all significantly greater than the expected 5.7405 except for the AUR (Met) codon family, suggesting selection against G-ending codons (i.e., large M_G3:U_). The exceptional AUR (Met) codon family has a tRNA with a CAU anticodon which would favor G-ending codons and is expected to be different.

**Table 3:** N_C_/N_U _ratios for NNY codons and N_A_/N_G _ratios for NNR codons in *Saccharomyces cerevisiae *(representing fungal mitochondrial genomes with translation table 3).

CF*	AA^†^	AC^‡^	N_Cod _^§^	N_C_/N_U_	CF*	AA^†^	AC^‡^	N_Cod _^§^	N_A_/N_G_
AAY	N	GUU	786	0.065	AAR	K	UUU	599	16.114
AGY	S	GCU	119	0.035	AGR	R	UCU	225	36.500
AUY	I	GAU	728	0.093	AUR	M	CAU	428	1.326
CAY	H	GUG	183	0.070	CAR	Q	UUG	165	8.706
GAY	D	GUC	264	0.052	GAR	E	UUC	220	9.000
UAY	Y	GUA	495	0.076	UGR	W	UCA	130	20.667
UGY	C	GCA	92	0.070	UUR	L	UAA	911	64.071
UUY	F	GAA	445	0.369					

Minimum				0.035					1.326
Mean				0.104					22.341
Std Dev				0.109					21.560

* CF: Codon family

^† ^AA: One-letter code of amino acid

^‡ ^AC: Anticodon

^§ ^N_Cod_: Number of codons in the codon family

The N_C_/N_U _ratio for the UUY (Phe) codon family is consistently greater than those of other NNY codon families (Tables 2, 3). One may suspect whether, for this particular codon family, there is a significant M_U3:G_. The rate of tRNA^Phe ^with anticodon 3'-AAG-5' dissociating from the UUU codon is about twice as high as that from the fully matched UUC codon [42]. Also, the tRNA^Phe ^misread CUC codons more than twice as often as CUU codons [42]. These two lines of evidence suggest that C3:G pair is much more favorable than U3:G pair, i.e., M_U3:G _for the UUY (Phe) codon family may indeed be substantially greater than M_C3:G_. Unfortunately, there has been no other similar studies on codon-anticodon pairing for other NNY codon families. One should also note that the tRNA^Phe ^in this study comes from *Escherichia coli*, and the result may not be applicable to fungal species.

The two fungal species using genetic table 16, i.e., *Spizellomyces punctatus *and *Rhizophydium sp. 136 *each have only a partial set of tRNA genes. Among NNY codon families in *S. punctatus*, only the GAY (coding for Asp) and UAY codon family (coding for Tyr) have an identifiable tRNA, with anticodons being GUC and GUA, respectively. The expected N_C_/N_U _and N_A_/N_G _ratios are 0.4536 and 2.2048, respectively, based on the nucleotide frequencies of non-coding sequences in the mitochondrial genome. The observed N_C_/N_U _ratios for GAY and UAY codon families are 0.1667 (= 40/240) and 0.2411 (= 81/336), respectively, suggesting no selection against U-ending codons (i.e., small M_U3:G_). In contrast, the N_A_/N_G _ratios are much larger, being 19.2308 (= 250/13) for the AAR codon family (coding for amino acid Lys) translated by a tRNA with a UUU anticodon and 11.1538 (= 145/13) for the CAR codon family (coding for amino acid Gln) translated by a tRNA with a UUG anticodon. These results suggest selection against G-ending codons (i.e., large M_G3:U_) There is no other NNR codon family with identifiable tRNAs in *S. punctatus*. The other species using translation table 16, *Rhizophydium sp. 136*, exhibits a similar pattern.

In short, results from fungal mitochondrial genomes are consistent with no selection against U-ending codons in NNY codon families but significant selection against G-ending codons in NNR codon families, indicating that M_U3:G _is smaller than M_G3:U_. These findings corroborate previous biochemical studies demonstrating that U3:G is energetically much more favorable than G3:U [2,23-26]. However, G3:U can be almost as good as A3:U when U is modified to xm^5^U [43].

The finding of a small M_U3:G _can explain puzzling observations in codon usage in fungal mitochondrial genomes. Take the tRNA^Ser ^translating the AGY codon family in the mitochondrial genome of *Ashbya gossypii *ATCC 10895 for example. The genome contains 31 AGU codons and no AGC codon. CAAH would have predicted an ACU anticodon with perfect base pair with AGU codons, but the observed anticodon is GCU consistent with WVH. Such an anticodon makes sense only if M_U3:G _is equal to M_C3:G_, i.e., a wobble G in a tRNA anticodon can pair with U just as good as with C in the third codon position.

Can this finding be generalized to nuclear genomes and the translation machinery in the cytoplasm? If M_U3:G _is generally small, then we have an answer to a puzzling observation that has long baffled molecular evolutionary geneticists. The UGY codon family (coding for Cys) in the nuclear genome of *S. cerevisiae *is translated by tRNA coded in four tRNA^Cys ^genes all with a wobble G at the tRNA anticodon. One would have predicted that UGC codons should be strongly preferred over UGU codons to avoid the wobble cost M_U3:G_. However, the observed numbers of UGC and UGU codons in *S. cerevisiae *CDSs is 13802 and 22873, respectively, opposite to the predicted trend. The bias is even stronger in highly expressed genes. For example, in the codon usage table of highly expressed genes distributed with the EMBOSS package [[44], Eyeastcai.cut], the numbers of UGC and UGU codons are 3 and 39, respectively. The unexpected codon usage bias (unexpected because the bias is in the wrong direction) becomes easy to understand if M_U3:G _is equal to M_C3:G_, i.e., there is no need to overuse the UGC codons to avoid M_U3:G _so AT-biased mutation in yeast (whose genome is highly AT-biased), which increases the frequency of U-ending codons, is not checked by counteracting selection.

If the finding of M_U3:G _<< M_G3:U _is applicable to nuclear genomes, then we predict that NNY codon families need only one type of tRNA (i.e., tRNA with a wobble G at the tRNA anticodon) for translation. In contrast, NNR codon families should ideally be translated by two different types of tRNAs, one with a wobble U for NNA codons and the other with a wobble C for NNG codons (to avoid the relatively high wobble cost of M_G3:U_). A corollary is that, if a NNY codon family is translated by only tRNA with a wobble G and NNR codon family by tRNA with a wobble U, then codon usage bias should be smaller in the NNY codon family (in which selection against G-ending codon is weak because of the small M_U3:G_) than in the NNR codon family (in which selection against U-ending codon is strong because of the relatively large M_G3:U_). Below we test these predictions with genomic data.

### NNY codon families are translated by one type of tRNA with a wobble G and NNR codon families are translated by two types of tRNA with a wobble U and a wobble C, respectively, in fungal nuclear genomes

We have inferred that a tRNA with a wobble G at its anticodon should be efficient not only in translating C-ending codons, but also in translating U-ending codons because of the small M_U3:G_. For this reason, only one type of tRNA with a wobble G should generally be sufficient in translating NNY codon families. In contrast, in NNR codon families, tRNA with a wobble U at its anticodon should be poor in translating G-ending codons because of the large M_G3:U_. Thus, the presence of G-ending codons in NNR codon families should favour the use of two types of tRNAs, one with a wobble U for translating NNA codons and another with a wobble C for translating NNG codons. In mitochondrial genomes with limited gene content, each codon family is generally translated by a single tRNA species. So this prediction cannot be tested. However, this prediction can be tested with nuclear genomes where the number of tRNA genes is not so limited as in mitochondrial genomes.

The prediction is strongly supported by results from the nuclear genome of *Saccharomyces cerevisiae *(Table 4). All NNR codon families are translated by two types of tRNAs with anticodons matching NNA and NNG codons, respectively, whereas all NNY codon families are translated by one type of tRNA with a wobble G at the tRNA anticodon (Table 4). This is consistent with the interpretation that the inference of M_U3:G _<< M_G3:U _derived from fungal mitochondrial genome is also applicable to fungal nuclear genomes.

**Table 4:** tRNAs translating two-fold codon families in the nuclear genome of *Saccharomyces cerevisiae*.

AA*	Codon^†^	T^‡^	F^§^
Arg	AGA	11	314
Arg	AGG	1	1
Gln	CAA	9	153
Gln	CAG	1	1
Glu	GAA	14	305
Glu	GAG	2	5
Leu	UUA	7	42
Leu	UUG	10	359
Lys	AAA	7	65
Lys	AAG	14	483
Asn	AAC	10	208
Asn	AAU	0	11
Asp	GAC	16	202
Asp	GAU	0	112
Cys	UGC	4	3
Cys	UGU	0	39
His	CAC	7	102
His	CAU	0	25
Phe	UUC	10	168
Phe	UUU	0	19
Ser	AGC	2	6
Ser	AGU	0	4
Tyr	UAC	8	141
Tyr	UAU	0	10

* Amion acid carried by tRNA

^† ^Codons forming Watson-Crick base pair with the anticodon of tRNA

^‡ ^Copy number of tRNA gene with anticodon matching the corresponding codon.

^§ ^Codon frequencies of highly expressed yeast protein-coding genes compiled in the Eyeastcai.cut file distributed with EMBOSS [44].

One may propose an alternative hypothesis for the observation that NNY codon families are translated by tRNAs with a wobble G whereas NNR codon families translated by tRNAs with a wobble C and a wobble U, respectively. The wobble A in some nuclear tRNAs is known to be converted to inosine or I for short [45-47] which may pair with A, C or U. If a tRNA translating an NNY codon family has its wobble G mutated to wobble A, then the wobble A may undergo the A→I conversion and misread NNA codons. For this reason, the wobble G→A mutation should be strongly selected against, which would explain the lack of wobble A in tRNA translating NNY codons.

This alternative hypothesis invoking the A→I conversion, while logically sound, was dismissed quite early after the discovery [45,47] that the A→I conversion is quite restrictive and occurs mainly at ACG or ARV anticodons (where R is the IUB code for A or G, and V is for A, G or C). Among tRNAs translating NNY codons, only tRNA^Phe ^has a middle A in the anticodon, and the rest do not have an R in the middle of the anticodon. This means that, for all NNY-translating tRNAs except for tRNA^Phe^, even if their regular wobble G mutates to A, the resulting wobble A will NOT be converted to inosine. Thus, the alternative hypothesis can only explain the avoidance of a wobble A in tRNA^Phe ^but cannot explain the avoidance of a wobble A in other NNY-translating tRNAs.

## Conclusion

In summary, our general hypothesis based on wobble costs allows the integration of the two conventional hypotheses (i.e., CAAH and WVH) on codon-anticodon wobble pairing. The integration leads to new ways of evaluating relative wobble cost of different wobble pairings. In particular, the finding that M_U3:G _is much smaller than M_G3:U _corroborates previous structural studies showing that U3:G is energetically more favourable than G3:U and leads to a better understanding of the translation efficiency mediated by codon and anticodon wobble pairing.
